# REThinking the role of the RET oncogene in breast cancer

**DOI:** 10.3389/fonc.2024.1427228

**Published:** 2024-08-01

**Authors:** Giuseppe Di Grazia, Chiara Conti, Sabrina Nucera, Gianmarco Motta, Federica Martorana, Stefania Stella, Michele Massimino, Mario Giuliano, Paolo Vigneri

**Affiliations:** ^1^ Department of Human Pathology “G. Barresi”, University of Messina, Messina, Italy; ^2^ Department of Clinical and Experimental Medicine, University of Catania, Catania, Italy; ^3^ University Oncology Department, Humanitas Istituto Clinico Catanese, Catania, Italy; ^4^ Center of Experimental Oncology and Hematology, Azienda Ospedaliera Universitaria (A.O.U.) Policlinico “G. Rodolico - S. Marco”, Catania, Italy; ^5^ Department of General Surgery and Medical-Surgical Specialties, University of Catania, Catania, Italy; ^6^ Department of Clinical Medicine and Surgery, University of Naples Federico II, Naples, Italy

**Keywords:** breast cancer, RET oncogene, targeted therapy, clinical trials, TKI - tyrosine kinase inhibitor

## Abstract

The REarranged during Transfection (RET) receptor tyrosine kinase plays a crucial role in the development of various anatomical structures during embryogenesis and it is involved in many physiological cellular processes. This protein is also associated with the initiation of various cancer types, such as thyroid cancer, non-small cell lung cancer, and multiple endocrine neoplasms. In breast cancer, and especially in the estrogen receptor-positive (ER+) subtype, the activity of RET is of notable importance. Indeed, RET seems to be involved in tumor progression, resistance to therapies, and cellular proliferation. Nevertheless, the ways RET alterations could impact the prognosis of breast cancer and its response to treatment remain only partially elucidated. Several inhibitors of RET kinase have been developed thus far, with various degrees of selectivity toward RET inhibition. These molecules showed notable efficacy in the treatment of RET-driven tumors, including some breast cancer cases. Despite these encouraging results, further investigation is needed to fully understand the potential role RET inhibition in breast cancer. This review aims to recapitulate the existing evidence about the role of *RET* oncogene in breast cancer, from its pathogenic and potentially prognostic role, to the clinical applications of RET inhibitors.

## 1 Introduction

The REarranged during Transfection (RET) oncogene was first discovered in 1980 and subsequently identified as a pivotal cancerogenic determinant for papillary and medullary thyroid carcinomas and other malignancies, paving the way for new diagnostic and therapeutic strategies (1).

The RET oncogene is involved in the regulation of cell growth and differentiation, playing a crucial role in embryonic development and in the maintenance of tissue homeostasis in adults (2).

RET rearrangements or mutations determine aberrant activation of its catalytic activity, contributing to tumor formation and progression (3). In particular, dysfunctional activation of RET signaling induces uncontrolled activity of the MAPK and PI3K/AKT/mTOR pathways, ultimately leading to cell proliferation (4).

Given the key role of the RET oncogene in cancer, its inhibition has represented an attractive therapeutic strategy. Indeed, several multi-tyrosine kinase inhibitors have been developed over the years and primarily tested in different tumor types, including thyroid carcinoma, renal cancer and hepatocellular carcinoma, where they currently represent the standard of care (5–7). More recently, selective RET inhibitors showed remarkable activity in multiple cancer types sharing RET rearrangements (8). Hence, selective RET inhibitors currently represent an agnostic therapeutic option for patients whose tumor harbor a RET fusion (9, 10).

With more than 2.3 million new diagnoses and 685.000 deaths in 2020, breast cancer (BC) represents a major health concern worldwide (11). Despite the significant advances achieved in the treatment of this disease, new and effective therapeutic options are continuously investigated and tested, in search for further improvement of disease outcome (12). Indeed, RET inhibition may represent an exploitable target across different BC subtypes.

Aim of this review is to provide an overview of the physiological and pathological functions of the RET oncogene, focusing on its role in BC biology. We reviewed the available pre-clinical and clinical evidence concerning RET pharmacological inhibition in this disease, also highlighting future challenges and perspectives for targeting this oncogene in the era of personalized medicine.

## 2 RET physiological and pathological functions

The REarranged during Transfection proto-oncogene is located on chromosome 10q11.2 and encodes for a transmembrane tyrosine kinase receptor (RTK) with an extracellular, a transmembrane and an intracellular domain (13). The extracellular region has four cadherin-like domains, a calcium binding site and a cysteine-rich domain. The intracellular region consists of the active tyrosine kinase domain flanked by two regulatory regions: the juxta-membrane domain and a C-tail (13).

**Figure 1:**
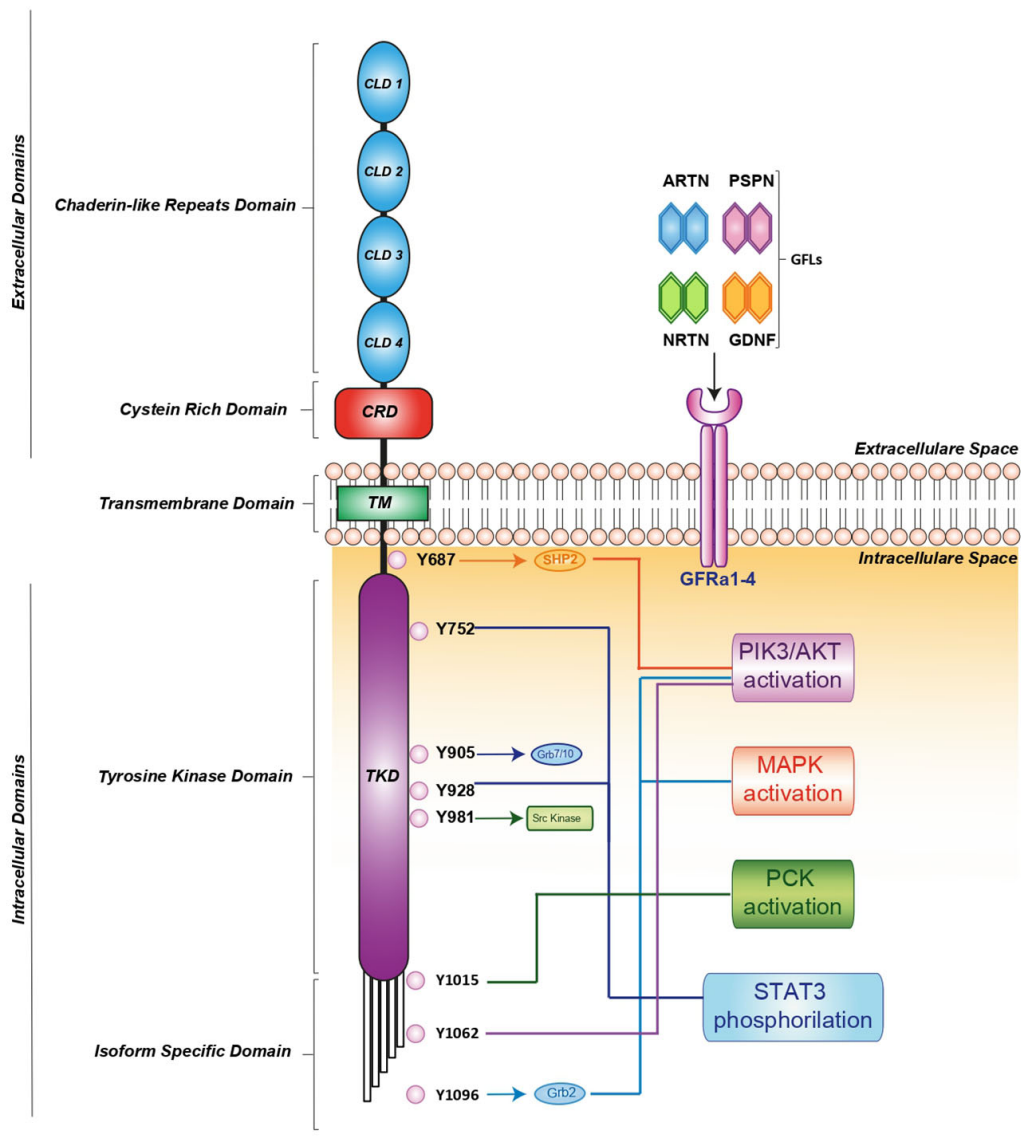
RET receptor structure, co-receptors, corresponding ligands and intracellular signaling pathway. RET activation is mediated by indirect interaction with one of four ligands: GDNF, ARTN, NRTN and PSPN linked to one of four GFRa1-4 co-receptors. The formation of the ligand/co-receptor/RET complex induces RET dimerization and triggers autophosphorylation at intracellular tyrosine residues leading the activation of downstream signaling pathways essential for cell growth, proliferation, survival, differentiation, or appetite control. The orange, light blue, blue and green arrows indicate the binding of adapter proteins. The light blue, blue, red, orange, green and violet lines indicate the activation of downstream signaling pathways. ARTN, Artemin; GDNF, glial cel-linederived neurotrophic factor; GFRa, GDNF family receptor-alfa; NRTN, Neurturin; PSPN, Persephin; RET, Rearranged during Transfection.

RET activation is triggered by glial cell line-derived neurotrophic factor (GDNF) family ligands (GFLs) such as GDNF, neurturin (NRTN), artemin (ARTN), or persephin (PSPN) that bind a glycosylphosphatidylinositol-anchored cell surface protein: GDNF family receptor-alfa (GFR-α) 1-4 (13). In turn, the GDNF-GFR α1 complex induces RET homodimerization, leading to auto-phosphorylation of its intracellular tyrosine residues (14, 15). These phosphorylated residues recruit several adaptor proteins, inducing the pleiotropic propagation of external stimuli. It has been clearly demonstrated that different residues activate distinct downstream pathways. For instance, RET-Y687 binds the SHP2 phosphatase, activating the PI3K/AKT pathway, while RET-Y752 and Y928 promote STAT3 phosphorylation, nuclear translocation and signaling. Moreover, RET-Y905 is critical for binding adaptor proteins Grb7/10 and RET-Y981 contributes to activation of the SRC kinase. Additionally, RET-Y1015 recruits phospholipase C-gamma (PLC-γ) thereby activating the protein kinase C (PKC) pathway, while RET-Y1062 recruits adaptor proteins which activate both the PI3K/AKT and MAPK pathways. Finally, RET-Y1096 binds Grb2 and activates the MAPK pathway (15) (**Figure 1**).

RET mRNA is overexpressed during the earliest phases of embryological development declining in the later phases of pregnancy. Indeed, a functional RET is necessary for renal embryogenesis and for the development of the sympathetic, parasympathetic and enteric nervous systems, allowing proper anatomical distribution of entero-endocrine and entero-chromaffin cells. Thus, intra-uterine RET loss may determine several malformations, including renal agenesia, severe kidney abnormalities, aberrant spermatogenesis or Hirschprung’s disease (16).

In adult life, high levels of RET can be found in the salivary glands, in the dopaminergic neurons of the substantia nigra and in the smooth muscle of the arterial wall, where they provide for proper functioning of the sympathetic nervous system. Lower RET expression can be observed in the heart, spleen, liver, kidneys, lungs, ovaries and testis (16).

Oncogenic *RET* activation mostly occurs because of chromosomal rearrangements or gene mutations that directly or indirectly activate its kinase domain (17).

Chromosomal rearrangements take place through the juxtaposition of 3’ *RET* sequences - encoding for the catalytic domain - with the 5’ sequences of other genes displaying a protein dimerization domain. Indeed, most *RET* fusions lack the transmembrane domain coding regions and give rise to a constitutively active protein. The most frequent rearrangements involve the coiled-coil domain containing 6 gene (*CCDC6-RET*), the nuclear receptor co-activator 4 gene (*NCOA4-RET*) and the kinesin family member 5B gene (*KIF5B-RET*) (15). *RET* fusions can be found in approximately 20% of papillary thyroid cancers, 1-2% of non-small-cell lung cancers (NSCLCs) and <1% of many other solid tumors, including ovarian, pancreatic, salivary and colorectal malignancies (18).

**Figure 2:**
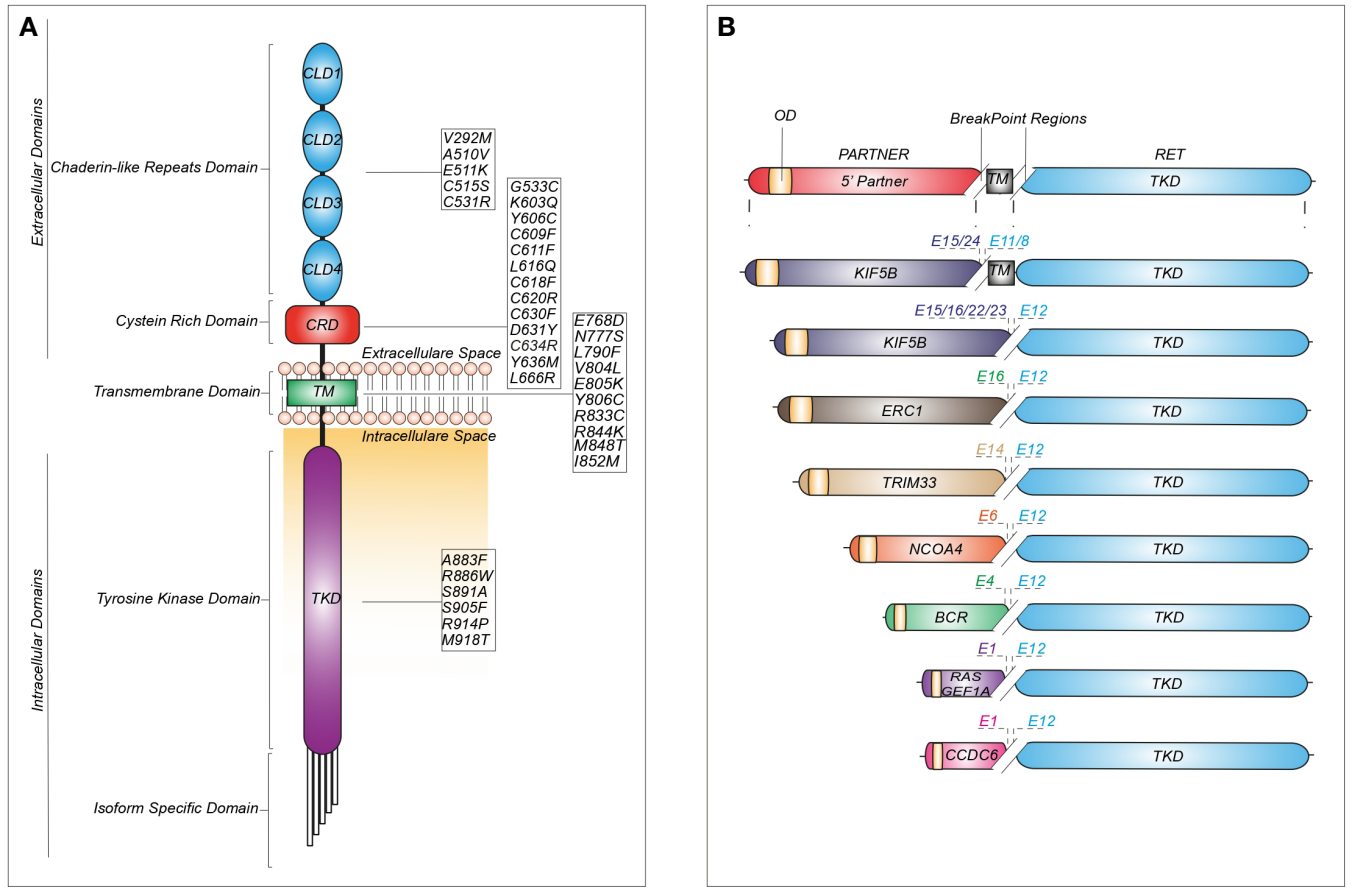
Schematic representation of recurrent RET mutations and rearrangements in cancer. **(A)** Structure of the RET protein reporting the most common mutations identified in cadherin-like repeats domains (CLDs), cystein-rich domain (CRD), transmembrane domain (TM) and tyrosine kinase domain (TKD). **(B)** RET fusions containing the most common upstream gene partners characterized by an oligomerization domain (OD) and different break-point regions dependent on the exon (E) involved in the generation of the chimeric junction.

Single nucleotide variants in the *RET* sequence can be either inherited or somatic (**Figure 2**).

Germline missense mutations are linked to autosomal dominant multiple endocrine neoplasia type 2 (MEN2), which is associated with an increased risk of medullary thyroid carcinoma (MTC), pheochromocytoma and other tumors (18). More than 95% of individuals with MEN2A display a germline mutation in *RET* exons 10 or 11, involving the cysteine-rich area of the extracellular domain. Indeed, these regions are prone to mutations which cause cysteine replacement with other amino acids. These modifications reduce the likelihood of generating intra-molecular disulphide-bonds, promoting instead inter-molecular covalent disulphide bonds between free cysteine residues of *RET* monomers, thereby increasing receptor dimerization and activation (19). Unlike MEN2A, MEN2B is associated with germline mutations in the kinase domain, such as the commonly reported substitutions M918T or A883F. Interestingly, the protein encoded by the RET-M918T variant can signal as a monomer, owing to increased ATP-binding affinity and altered protein conformation, leading to loss of kinase autoinhibition (20).

Somatic *RET* mutations are found in 65% of all sporadic MTCs. The majority of these tumors present the RET M918T mutation, although additional somatic substitutions, such as E768D and A883F, have also been described. Less frequently, *RET* mutations can be found in sporadic pheochromocytoma (15%) (20).

The increasing use of NGS platforms has uncovered both classical and novel *RET* mutations in other cancers, including RET-C634R in breast carcinoma, RET-E511K in endometrial and Merkel-cell carcinomas, RET-M918T in a paraganglioma and atypical lung carcinoid and RET-V804M in colorectal carcinoma, meningioma, gastrointestinal stromal tumors (GIST) and hepatoma (9) (**Figure 2**).

## 3 RET as an oncogene in breast cancer

**Figure 3:**
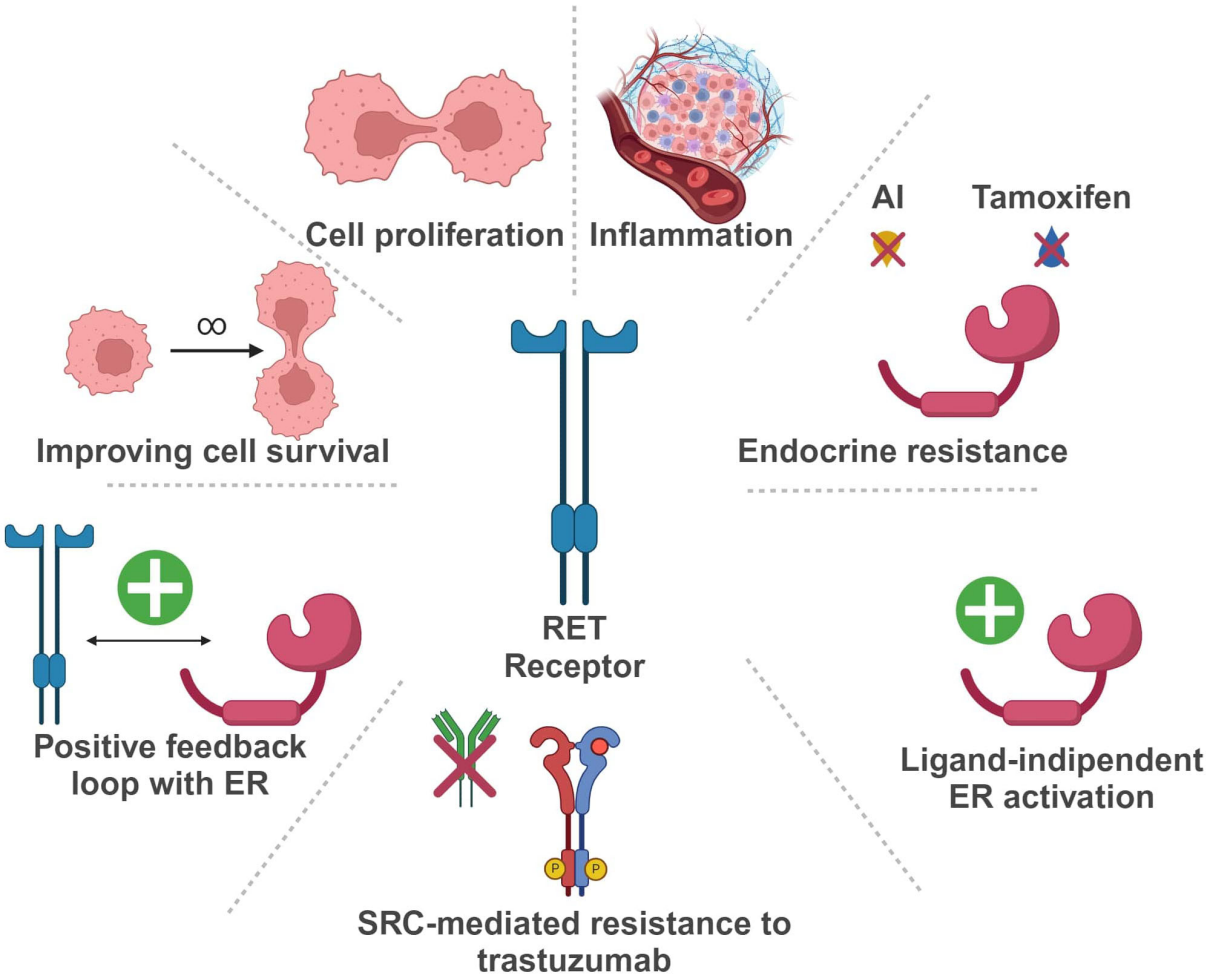
Schematic representation of RET oncogene role in Breast Cancer (Created with BioRender.com). RET, Rearranged during Transfection; ER, estrogen receptor; AI, aromatase inhibitor.

In the last years, the potential role of *RET* in BC development and progression has been extensively investigated (**Figure 3**).

In BC, *RET* is more frequently overexpressed than rearranged or mutated. Indeed, high levels of *RET* RNA, with or without gene amplification, can be found in up to 40-60% of breast tumors, usually correlating with increased protein expression (21–25).

More in detail, in ER positive (ER+) BC, *RET* overexpression seems to be involved in tumorigenesis and resistance to endocrine therapy (26–30). In a study by Gattelli et al., chronic overexpression of wild-type *RET* (isoform 51) in the mammary gland promoted the development of ER+ BC in a transgenic mouse model (22). These tumors harbored intense MAPK and PI3K/AKT/mTOR signaling, underlying the relevance of these pathways in RET-driven BC, as reported in additional studies (24, 27, 30, 31). According to Wang et al., exposure of BC cells to estrogen enhances RET expression through ER and FOXA1 transcriptional activity, leading to the activation of downstream pathways and eventually promoting cell proliferation (32).

RET hyperexpression may also determine estrogen-independent activation of the estrogen receptor (ER)-α, leading to an endocrine resistant phenotype (26, 27, 33). Moreover, autocrine production of RET ligands, such as GDNF, by ER+ BC cells may be responsible for RET-mediated endocrine resistance, rather than RET overexpression itself (34). However, high levels of RET and GDNF in ER+ BC cells seem to be associated with estrogen-independent signaling activation via NTRK, KRAS and PI3K, and these features seemingly correlate with inferior survival outcomes (35). In turn, ER activation can upregulate both *RET* and *GFR-α1*, due to the presence of estrogen-responsive elements (EREs) in their promoters (36, 37). Other evidence supports a role for RET in the development of endocrine resistance. In an *in-vitro* endocrine resistant BC model, the GDNF/RET axis was strongly active and promoted cell survival (33, 38). Hyperactivation of this pathway may determine estrogen-independent ER functioning and promote the transcription of genes related to the immune response, such as *STAT1* and *STAT3* (22). Additionally, GDNF/RET signaling seems to induce a positive feedback loop with early growth response-1 (EGR1) that can cause endocrine resistance through the activation of cyclin D1 (39).

Zheng and colleagues described another positive feedback loop involving BRD4/ER-α-RET-ER-α in ER+ BC cell lines (40). In detail, estrogen receptor super-enhancers (ERSEs) seem to promote ER-α-induced carcinogenesis, regulating transcription of ER target genes, such as *RET*. Moreover, the bromodomain protein BRD4, which is a massive regulator of ERSEs, controls *RET* activation through ERSEs itself. On the other hand, RET activation induces the up-regulation of the RAS/RAF/MEK2/ERK/p90RSK/ER-α cascade, leading to a loop activation of ER-α, a crucial event for ER-α-induced gene transcription and development of a malignant phenotype (40).

On a different note, in ER+ BC cells endocrine therapy may exert a paradoxical effect on the GDNF/RET axis, increasing RET expression at both the RNA and protein level, a mechanism that seemingly relies on pro-inflammatory mediators (24, 41). Indeed, it has been reported that both fulvestrant and aromatase inhibitors can increase inflammatory cytokines levels (e.g. IL-6) (24, 41). Furthermore, the *RET* promoter displays binding sites for STAT and NFKB, that can stimulate IL-6 expression. In line with these observations, adding an anti-IL-6 antibody in BC cell culture reduces RET expression. These data reinforce the hypothesis that RET signaling can mediate endocrine resistance through inflammation mechanisms (31, 33).

Given its involvement in endocrine resistance, RET overexpression has controversially been proposed as a negative prognostic biomarker in ER+ BC. In a cohort of 93 ER+ BC patients, individuals carrying a *RET* polymorphism associated with lower *RET* expression (rs2435357C>T) displayed longer survival (42). Consistently, several reports have described an association between RET overexpression and a worse prognosis (31, 32). However, RET hyperexpression by immunohistochemistry did not predict inferior survival in two large cohorts of ER+ BC (32, 43).

*RET* overexpression can also be present in HER2-enriched (HER2+ve) and triple negative (TN) BC (6, 38). In HER2+ tumors, RET can cause resistance to anti-HER2 therapies, such as trastuzumab. Indeed, GDNF/RET signaling can reduce trastuzumab-induced apoptosis, eventually promoting cell survival and treatment resistance. In a xenograft model, the addition of recombinant GDNF (rGDNF) in cultures stimulates tumor growth in both trastuzumab-sensitive and -resistant cell lines (38). This effect is reversible upon SRC inhibition, suggesting a potential role for SRC in RET-driven resistance to trastuzumab (38).

The role of *RET* hyperexpression in TNBC is not fully elucidated. However, RET-directed TKIs seem to inhibit TNBC growth, providing preclinical evidence for the use of RET inhibitors in this subtype of breast carcinomas (6).

Besides *RET* overexpression, other genomic aberrations have been reported in BC. *RET* fusions are exceedingly rare (0.1%) but represent a potential therapeutic target for BC patients (44). The most common rearrangements are *CCDC6-RET*, *NCOA4-RET* and *RasGEF domain family member 1A (RASFGEF1A)-RET* (45). Additionally, new fusion partners have been recently identified, such as *ELKS*/*RAB6*-*interacting*/*CAST family member 1* (*ERC1*)*-RET, zinc finger protein 485* (*ZNF485*)*-RET* and *Sperm Antigen with Calponin Homology and Coiled-Coil Domains 1 Like (SPECC1L)-RET* (45, 46). Similarly, *RET* SNVs have been anecdotally described in breast tumors (0.2%) (17). They are usually missense mutations, such as C634R and M918T (responsible for MEN2A and MEN2B, respectively). Other substitutions, such as E511K, C611R, C620F, L633V, C634F and T636M, involve the extracellular domain, while V804M occurs in the kinase domain (45). These SNVs have been found more frequently in metastatic sites, rather than in primary tumors. Several *in vitro* studies have defined the pathways activated by these alterations that include MAPK, PI3K/AKT, mTOR, FAK and JAK/STAT (47). Other mutations, such as M918T, can maintain STAT3 constitutively active (48). Lastly, alterations involving non-coding *RET* sequences (e.g., promoter regions) that may influence the expression of TK domain coding exons have also been reported in BC (49–51), but their biological significance is still uncertain.

## 4 Targeting RET in breast cancer

### 4.1 Pre-clinical evidence

According to preclinical evidence, RET represents an actionable target in many solid tumors, including BC, and different RET inhibitors have been tested both *in vitro* and in xenograft models. However, most of the evidence available so far, regards mainly multi-kinase inhibitors, whose anti-RET activity cannot be easily assessed.

Among these, sunitinib (*N*-[2-(diethylamino)ethyl]-5-[(*Z*)-(5-fluoro-2-oxo-1*H*-indol-3-ylidene)methyl]-2,4-dimethyl-1*H*-pyrrole-3-carboxamide) reduced MAPK/ERK pathway activity in RET-expressing BC cell lines (7). Similarly, treatment with vandetanib reduced RET phosphorylation and activation, promoting tumor regression in BC patient-derived xenografts (PDXs) (6).

More recently, cabozantinib (1-N-[4-(6,7-dimethoxyquinolin-4-yl)oxyphenyl]-1-N’-(4-fluorophenyl)cyclopropane-1,1-dicarboxamide) has been tested in RET overexpressing cell cultures and PDXs from resected brain metastases, determining a reduction of RET phosphorylation and inhibiting tumor growth (52).

RET inhibitors have also been tested in association with endocrine therapy in ER+ BC, with controversial results. The combination of both sunitinib or vandetanib and tamoxifen significantly reduced tumor growth *in vivo* (28, 30, 53). On the other hand, the addition of tamoxifen, letrozole or fulvestrant to NVP-AST487 (a multi-kinase RET-targeting inhibitor) did not show an additional benefit in xenograft models (24). However, these combinations seem to decrease disease metastatic index and tumor dissemination (24).

Limited preclinical evidence has been generated in BC models harboring *RET* rearrangements with the available evidence indicating that cabozantinib reduces the proliferative potential of breast cancer cells displaying the *NCOA4-RET* fusion (52).

Finally, an antibody-drug conjugate (ADC) encompassing a fully human anti-RET antibody (Y087) and the microtubule inhibitor maytansine (DM1) has been developed and tested both *in vitro* and *in vivo*. The compound showed signs of activity in BC xenograft models, with preliminary safety studies in primates suggesting neuropathy as the main on-target toxicity (54).

### 4.2 Clinical evidence

Given the encouraging preclinical results, RET inhibitors have been tested in BC patients, mostly in early phase trials (**Table 1**). Results are already available from studies with either multi-kinase inhibitors (anlotinib, cabozantinib, lenvatinib, vandetanib) or RET-specific inhibitors (selpercatinib), with several other trials still ongoing (**Table 2**).

**Table 1:** Published clinical trials with RET-inhibitors including breast cancer patients.

Disease	Trial Identifier	Pts	Receptors status	Intervention	Phase	Endpoints and Results
Advanced Solid Tumors	NCT00940225	45	ER+/HER2- n=35 ER+/HER2+ n= 7 ER+/HER2u n=1 TN n=2	Cabozantinib	II	ORR (13.6%) PFS (4.3 m) OS (11.4 m)
Breast cancer	NCT01441947	52	ER+/PgR+ n=40 ER+/PgR- n=12	Cabozantinib	II	Bone scan RR (38.5%) DCR (50%) OS (19.6 m) PFS (4.3 m)
Breast cancer	NCT02562118	47	ER+/PgR+ n=40 ER+/PgR- n=6	Lenvatinib + Letrozole	Ib/II	ORR (23.3%)
Breast cancer	NCT00811369	129	HR+ n=123 HER2+ n= 6	Vandetanib + Fulvestrant	II	uNTx PFS (5.8 m) OS
Breast cancer	NCT04002284	26	HR+ n=16 HR- n= 10	Anlotinib	II	ORR (15.3%) PFS (5.22 m) DCR (80.7%)

Pts, number of patients; HER2u, HER2 unknown; PFS, progression-free survival; ORR, overall response rate; DFS, disease-free survival; OS, overall survival; uNTx, urinary N-telopeptide; Bone scan RR, Bone scan response rate.

**Table 2:** Ongoing clinical trials with RET-inhibitors enrolling breast cancer patients.

Disease	Trial Identifier	Intervention	Regimens	Phase	Endpoints
HR+/HER2- breast cancer	NCT05181033	Lenvatinib + Letrozole vs Fulvestrant	14 mg daily + 2.5 mg daily vs 500 mg day 1 every 4-weekly cycle	II	PFS, ORR, CBS, OS
HR+/HER2- breast cancer	NCT05286437	Lenvatinib + Letrozole + Pembrolizumab	14 mg daily + 2.5 mg daily + 400 mg day 1 every 6-weekly cycle	II	ORR, PFS, DOR, CBR, OD
HR+/HER2- breast cancer	NCT05075512	Anlotinib + Fulvestrant	12 mg once daily on days 1-14, every 21 days + 500 mg day 1 every 4-weekly cycle	II	PFS, ORR, CBR, OS, AE
Advanced solid tumors RET fusion + solid tumors MTC	NCT03157128	Selpercatinib	20 mg once daily or 20-240 mg twice/160 twice	I/II	ORR, OS, PFS, DOR

PFS, progression-free survival; ORR, overall response rate; DFS, disease-free survival; OS, overall survival; CBR, clinical benefit rate; DOR, duration of response; AE, adverse event.

#### 4.2.1 Non-selective multi-kinase inhibitors

Anlotinib (1-[[4-[(4-fluoro-2-methyl-1H-indol-5-yl)oxy]-6-methoxyquinolin-7-yl]oxymethyl]cyclopropan-1-amine) is a tyrosine kinase inhibitor with several targets, including RET, FGFR, c-KIT, PDGFR, and VEGFR (55, 56). The efficacy and safety of this inhibitor has been evaluated in a Chinese population with advanced solid tumors (55). In a single-arm phase II trial, 26 HER2- metastatic BC patients, unselected for the presence of *RET* alterations, received anlotinib after failing standard treatment options (56). Objective response rate (ORR), the primary endpoint, was 15.4%, while median PFS was 5.22 months. Overall, anlotinib activity was superimposable in ER+ and TNBC patients. Treatment was well tolerated with grade 3-4 adverse events (AEs) mostly ascribed to hypertension (26.9%) and hand-foot syndrome (3.8%) (56).

The multi-kinase inhibitor cabozantinib targets RET, MET, VEGFR2 and other receptor tyrosine kinases such as FLT3 and c-KIT (5). In a single-arm phase II trial, cabozantinib, at the dose of 100 mg or 60 mg once daily, showed clinical activity in 52 ER+ metastatic pre-treaded BC patients with bone metastases (with or without extra-osseous metastases) (57). Bone scan response rate, the primary endpoint, was 38.5%, while disease control rate (DCR) was 50%. Median PFS and OS were 4.3 and 19.6 months, respectively. No difference emerged between patients with bone-only and extra-osseous disease. Approximately 80% of patients receiving cabozantinib at 100 mg required a dose reduction. Most common adverse events were fatigue, hypertension, diarrhea and nausea (57). In another phase II trial conducted in different tumor types, cabozantinib was administered for a 12-week, open-label lead-in period. Patients with partial response at week 12 were kept on open label cabozantinib, while those with stable disease were randomized to cabozantinib or placebo. At the time of disease progression, treatment was unblinded and patients on cabozantinib discontinued it, while those on placebo resumed the TKI until a new progression occurred. Objective response rate (ORR) for the 12-week lead-in stage and PFS after randomization were the co-primary endpoints. Among the 45, heavily pretreated, BC patients enrolled (43 ER+ve, 2 TN) ORR at 12 weeks was 13.6% and DCR 46.7%. Median PFS was 4.3 months and median OS was 11.4 months. Most frequent adverse events of all grades were fatigue, palmar-plantar erythrodysesthesia, nausea and diarrhea (58). A published report describes the case of a 63-year-old woman with recurrent ER+/HER2+ BC and a *NCOA4-RET* rearrangement that achieved a clinical and radiological response to the combination of cabozantinib, trastuzumab and exemestane (45).

The multi-kinase inhibitor foretinib (1-*N*’-[3-fluoro-4-[6-methoxy-7-(3-morpholin-4-ylpropoxy)quinolin-4-yl]oxyphenyl]-1-*N*-(4-fluorophenyl)cyclopropane-1,1-dicarboxamide) has been tested, in combination with lapatinib, in a phase Ib trial that enrolled 19 patients with advanced HER2+ BC. However, the TKI association showed limited activity (median PFS 3.2 months), and foretinib development has been halted (59).

Lenvatinib (4-[3-chloro-4-(cyclopropylcarbamoylamino)phenoxy]-7-methoxyquinoline-6-carboxamide) is another multi-kinase inhibitor with several targets, such as RET, FGFR, c-KIT, PDGFR, and VEGFR (60–62). In a phase Ib/II trial the combination of lenvatinib with letrozole was evaluated in 47 post-menopausal women with pre-treated advanced ER+/HER2- BC (63). Twenty-three percent of patients presented an objective response, with a median duration of 6.9 months and a median time-to-progression of 6.2 months. The potential correlation between RET expression by immunohistochemistry and clinical benefit was investigated. Despite a trend toward an increased efficacy in patients with higher RET expression, the association was not statistically significant.

Vandetanib (N-(4-bromo-2-fluorophenyl)-6-methoxy-7-[(1-methylpiperidin-4-yl)methoxy]quinazolin-4-amine) is a multi-kinase inhibitor with activity against and RET, EGFR1 and VEGFR2. A randomized phase II trial investigated the use of vandetanib or placebo with fulvestrant in 129 postmenopausal ER+ metastatic BC patients, with bone only or bone-predominant disease (64). Reduction of urinary N-telopeptide of type 1 collagen (uNTx) was the primary outcome of this study. Secondary outcomes included PFS, OS, pain response and number of skeletal-related events (SREs). The trial failed to show any statistically significant difference between treatment arms for both the primary and secondary endpoints, indicating no advantage of adding vandetanib to fulvestrant in this population (64).

#### 4.2.2 Selective inhibitors

Selpercatinib (6-(2-hydroxy-2-methylpropoxy)-4-[6-[6-[(6-methoxypyridin-3-yl)methyl]-3,6-diazabicyclo[3.1.1]heptan-3-yl]pyridin-3-yl]pyrazolo[1,5-a]pyridine-3-carbonitrile) is a selective inhibitor of wild-type, mutant and re-arranged RET. The efficacy of this drug has been explored in a phase I/II, multicenter, open-label, multicohort clinical trial (LIBRETTO-001) in patients with tumors harboring *RET* alterations (65, 66). Two patients with BC and *RET* fusions were enrolled in this trial, experiencing a partial and a complete response, respectively. The patient achieving a complete response was a 46-year-old pre-menopausal Japanese woman, with multiple nodal and lung metastases from ER+ BC at baseline, progressing to first line therapy with tamoxifen and goserelin (67). Next generation sequencing analysis performed on tumor tissue detected the *CCDC6-RET* fusion. Upon selpercatinib initiation, the patient experienced a rapid clinical improvement, along with a partial response, followed by a complete response after 3 months of treatment (67).

Another report described the case of a 36 years-old Chinese woman with heavily pretreated TNBC, displaying a *CCDC6-RET* fusion and achieving a partial response with the RET selective inhibitor pralsetinib (N-[(1S)-1-[6-(4-fluoropyrazol-1-yl)pyridin-3-yl]ethyl]-1-methoxy-4-[4-methyl-6-[(5-methyl-1H-pyrazol-3-yl)amino]pyrimidin-2-yl]cyclohexane-1-carboxamide) (68).

Several clinical trials are evaluating the efficacy of RET inhibitors in monotherapy or in combination with other agents in BC patients. Most of these early phase trials are testing a combination of multi-kinase inhibitors and hormonal therapy in patients with metastatic ER+/HER2- BC, progressing after a previous line of endocrine therapy.

## 5 Conclusions and future perspectives

The intricate role of the *RET* oncogene in BC biology has been extensively explored since the early nineties, with most findings generated in the ER+ subtype (3, 33, 34). As this body of evidence shows, in BC RET is frequently overexpressed rather than rearranged or mutated (21, 35, 69). Still, thus far a prognostic or predictive role for RET hyperexpression in breast malignancies has not been demonstrated.

In ER+ BC patients, alterations of RET signaling contribute to the onset and maintenance of endocrine resistance (29, 34, 39). Whether these alterations are also implicated in lack of response to other therapies, such as cyclin dependent kinase 4/6 (CDK4/6) inhibitors or ADCs, is unknown, and could represent a field for future investigations.

Data from clinical trials testing non-selective RET inhibitors in BC, either as monotherapy or in combination with endocrine therapy, are conflicting and results from ongoing trials are awaited (57, 60, 64, 70).. Among these, a phase II study is comparing the combination of lenvatinib and letrozole to fulvestrant in advanced ER+ BC patients who progressed to first line endocrine therapy plus a CDK4/6 inhibitor (NCT05181033). Another phase II trial is testing lenvatinib, pembrolizumab and letrozole in endocrine-resistant advanced BC (NCT05286437). The association of lenvatinib and pembrolizumab has proven effective in multiple cancer types, including some scarcely sensitive to immune-checkpoint inhibitors monotherapy (e.g., mismatch repair-proficient endometrial cancer) (71). Hence, a rationale exists to test this combination in ER+ BC. In general, a major pitfall of past and ongoing studies of non-selective RET inhibitors in BC is the lack of a predictive biomarker that could inform patient selection.

In the last years, the search for actionable molecular drivers has led to major breakthroughs in the treatment of several malignancies and to the definition of so-called agnostic targets, which offer the opportunity to tailor patient treatment to the disease molecular profile rather than to the tumor site of origin (72, 73). This is the case for *NTRK* rearrangements that can be addressed by specific inhibitors, and for tumor mutational burden (TMB) or mismatch repair deficiency (MMRd), that are predictive of immunotherapy efficacy (74–76). More recently, *RET* rearrangements have also been claimed as agnostic biomarkers and in 2022 the Food and Drug Administration approved the selective RET inhibitor selpercatinib for the treatment of patients with advanced solid tumors with *RET* gene fusions after the results of the phase I/II LIBRETTO trial (10).

Moreover, ADCs targeting RET or GFRA1, may represent another innovative approach in the future, although only preclinical evidence is available so far (54, 77).

Despite being exceedingly rare, *RET* rearrangements represent an invaluable therapeutic possibility for BC patients. Published reports confirm the activity of selective RET inhibitors across different BC subtypes in patients with advanced disease, progressing to several lines of standard treatments (9, 10). *RET* point mutations may also predict response to selective inhibitors, as already demonstrated in medullar and papillary thyroid cancer (65). However, whether the same activity is retained in BC displaying *RET* mutations has yet to be demonstrated.

Importantly, RET inhibitors, especially multi-kinase compounds, may induce different and potentially severe toxicities, such as hypertension and hemorrhagic or thrombotic events (7, 9, 10, 55, 56, 58, 59, 63, 64). Future studies evaluating these drugs must take into account strategies to mitigate and manage toxicity, including dose reduction or intermittent schedules, cardiological monitoring, evaluation of concurrent medications and comorbidity.

Overall, clinical application of RET inhibitors in BC is still in an early stage of development. Most of the existing evidence derive from small studies, and no recommendation exists about the *RET* testing and its timing in BC patients. Since RET inhibitors represent an off-label option for BC patients in most countries, with the exception of the United States where selpercatinib has received an agnostic indication, *RET* alterations could be tested in patients with advanced BC after progression to standard options, to check for potential eligibility in clinical trials.

Future researches should focus on: i) mechanistic studies elucidating the precise molecular mechanisms by which RET alterations drive BC progression and therapy resistance; ii) combinations between RET inhibitors and other compounds such as hormonal treatments and immunotherapies; iii) identification of reliable predictive biomarkers,to select patients who are most likely to benefit from RET-targeted therapies.

Understanding the role of the *RET* oncogene in BC biology may increase our ability to exploit its therapeutic potential in the era of personalized medicine. Future studies should elucidate the prognostic and predictive role of *RET* alterations in BC, with the aim of expanding the proportion of patients who may benefit from RET inhibition, eventually integrating them into the therapeutic armamentarium for BC patients.

## Author contributions

GDG: Conceptualization, Writing – original draft. CC: Writing – original draft. SN: Writing – original draft. GM: Methodology, Writing – original draft. FM: Conceptualization, Methodology, Writing – review & editing. SS: Writing – review & editing. MM: Writing – review & editing. MG: Supervision, Writing – review & editing. PV: Supervision, Writing – review & editing.
